# Untoward immune effects of modern medication

**DOI:** 10.7555/JBR.37.20230071

**Published:** 2023-12-18

**Authors:** Daohong Chen

**Affiliations:** Research Institute, Changshan Biochemical Pharmaceutical, Shijiazhuang, Hebei 050800, China

**Keywords:** immunotoxicology, immune-related adverse events, anti-drug antibody

## Abstract

Immune-related adverse events (irAEs) represent an increasingly concerning challenge in the assessment of biopharmaceutical products. In contrast to historically rare allergic reactions associated with small chemical drugs, contemporary biotherapeutics exhibit a significantly higher morbidity of irAEs, because of their complex structure and comprehensive mechanisms of action. While the immunogenicity of protein-based compounds is associated with the induction of anti-drug antibodies, the pathogenesis of irAEs in advanced biologics, such as cell and gene therapy, remains to be further delineated. In the current study, I present an updated profile regarding the untoward immune effects of medications, covering various material categories systematically, with the underlying mechanisms to inspire risk mitigation in biopharmaceutical development and application.

## Introduction

As an official paradigm for regulatory approval, the clinical assessment of pharmaceutical products is principally based on comprehensive evidence of efficacy and safety that can be substantially affected by immune-mediated side effects. Historically, immunogenicity-induced adverse reactions represent only a minimal portion of the drug-associated toxic profiles because of their rare incidence^[1–2]^. However, in the contemporary therapeutic landscape, concerns over untoward immune effects escalate, posing a significant challenge to medical practice and pharmaceutical development, especially with the advent of biological medicine characterized by their complex structures, that offer unique benefits in addressing unmet clinical needs^[2–3]^. In this scenario, it has been revealed that peptide/protein formulations can be antigenic, prompting the host immune system to generate anti-drug antibodies (ADAs) during patient treatment courses^[3]^. Moreover, antibody/cell-based medications exert their therapeutic effectiveness frequently through modulating the human immune system, which may simultaneously raise the possibility of immune-mediated untoward reaction^[4–5]^.

It is increasingly recognized that human immune responses to pharmaceutical products have the potential to affect clinical pharmacodynamics, pharmacokinetics, efficacy, and safety in the treated patients^[3]^. Additionally, the presentation of immune-related adverse events (irAEs) varies considerably in terms of severity grades, including the manifestations such as rash, fever, organ damage, anaphylaxis, *etc*.^[1,3,6]^. To date, while having inspiring the development of numerous novel innovative medications, the interdisciplinary breakthroughs in biomedical sciences in recent years have significantly contributed to a better understanding of the cellular and molecular mechanisms behind drug immunity-driven adverse events^[3,6]^. In this context, official guidelines regarding the assessment of immunogenicity risks, as a part of toxicology reports, have recently been announced by the major regulatory agencies for large molecular pharmaceutical products, such as therapeutic proteins and heparin formulations, before approval for human use^[3–4,7]^.

**Figure 1:**
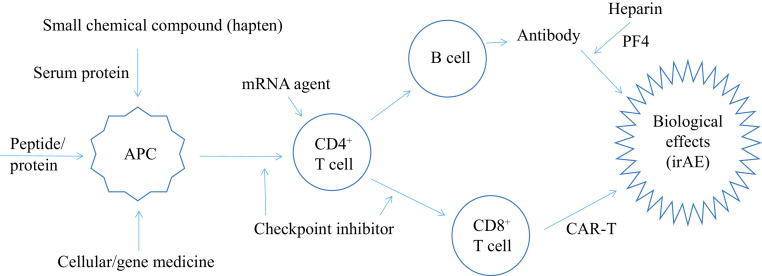
A machanism summary for irAEs of medication.

While novel categories of medications, such as RNA formulations and viral vector-bearing agents, increasingly enter the landscape of the medical market, unwanted immunogenicity is continuously evolving to face clinical practice, pharmaceuticals, and regulatory assessment^[4,8]^. Moreover, along with real-world data accumulated through post-marketing surveillance, the emerging aberrant immunity-linked adverse events need to be timely monitored for certain existing drugs^[9]^. Therefore, the current study presents an updated profile of untoward immune effects for major representative types of biomedical products, with the pathogenesis mechanisms to inspire pharmaceutical mitigation (***Fig. 1***).

## Small chemical compound

The accumulation of knowledge regarding immune-mediated adverse reactions began with the understanding of the drug allergic phenotype triggered by low molecular weight medications, such as penicillin and sulfonamides^[1,10]^. It was hypothesized that these drugs became immunogenic to stimulate a host response by binding to serum proteins according to the hapten theory or by modifying the surface receptors of immune cells. The underlying pathogenesis pathways involving immunoglobulin E (IgE), IgG, IgM, drug-antibody complexes, and T cells, have led to various types of allergic events, such as anaphylaxis and delayed hypersensitivity reactions in the clinic^[1,6]^. While the medical presentation of chemical drug allergy are highly heterogeneous from skin lesions to organ damages, the list of etiologic compounds has continuously been extending to involve viral and kinase inhibitors among other emerging agents in recent years^[1,6,11]^. For instance, ibrutinib and idelalisib were sometimes observed to induce immune pathology-associated interstitial nephritis and pneumonitis, respectively^[11–12]^. Fresh insights into pathogenesis have revealed a link between specific human leukocyte antigen genes and differential chemical compounds. These antigen genes may serve as biomarkers in addition to skin tests to predict clinical risks of immunogenicity. Intriguingly, while cluster of differentiation 4 (CD4)^+^ T lymphocytes play a role in initiating drug-induced immunogenicity, CD8^+^ T cells contribute to the inflammatory organ pathology^[6,13–14]^. In medical practice, the mainstream approach for circumventing the challenge is to avoid the allergic agents but instead take alternative drugs without cross-reactivity^[1,10]^. On the other hand, from a pharmaceutical consideration, it has been proposed that the improved quality control of therapeutic products, such as the diminished antigenic epimers or allergy-linked structural elements in antibiotic development and manufacturing, helps down-regulate the interaction with IgE and can mitigate risks of drug immunogenicity^[15–16]^.

## Peptide

Traditionally, therapeutic peptides represent the purified or recombinant versions of endogenous proteins that have important physiological activities and are normally circulating at low concentrations in the human body, including hormones, growth factors, clotting molecules, *etc*.^[3,17]^. Nonetheless, studies have shown that repeated injections of peptide medications over a long term can break immune tolerance to these self-antigens and thus induce specific lymphocyte activation with an elevated ADA^[17–18]^. In this sense, insulin antibodies (IAs) have been identified in diabetic patients for regular insulin treatments, and are associated with exogenous insulin antibody syndrome (EIAS) that is characterized by insulin resistance with hyperglycemia or hypoglycemia. Namely, the IAs with a low affinity/high capacity result in a postprandial hyperglycemia and a nocturnal hypoglycemia, meanwhile the IAs with a high affinity/low capacity induce EIAS with a severe insulin resistance^[19]^. Likewise, recombinant human erythropoietin (EPO) serves as an outstanding therapeutic peptide medicine for patients with low hemoglobin caused by certain serious illnesses, such as chronic renal disease and cancer. However, a long-term application of exogenous EPO may activate specific T cells, and thus stimulate the neutralizing ADAs that cross-react with endogenous EPO, leading to pure red cell aplasia consequently^[20]^. While the biological medication with recombinant factor Ⅷ confers a specific efficacy for the patients with hemophilia A, the neutralizing antibodies were stimulated in 20%–30% of the treated patients, thus resulting in the replacement therapy inefficient and even increasing mortality^[21]^. In terms of mitigating measures for the above-mentioned peptide drugs and beyond, one possibility is to stop the antigenic agents, shifting to alternative medical options if available in the clinic^[3]^. Regarding pharmaceutical optimization in parallel, it has been insightfully explored to de-immunize the therapeutic proteins through advanced formulations, post-translational modifications, or/and a selective point mutation strategy to remove those crucial sites binding to human leukocyte antigen in the sequence of amino acid residues with preservation of due biologic effects^[18,20–21]^.

## Antibody

The success of targeted therapy driven by antibody technology has revolutionized clinical management in numerous aspects^[5]^. However, it is also recognized that antibody therapy may raise the morbidity of unwanted immune response-mediated side effects because of the immunity-modulating nature of antibody function and the potential antigenic activity of exogenous immunoglobulins that are characterized by a high molecular weight as well as a complex structure^[3–4]^. Therefore, the ADAs have been identified in up to 60% of autoimmune patients on the treatment of antibodies against tumor necrosis factor-α (TNF-α), including adalimumab, and these ADAs were largely neutralized^[22–23]^. Consequently, the anti-TNF-α inhibitor ADAs led to the reduced efficacious outcomes and higher rates of relevant side effects in the clinic^[23]^. On the other hand, anti-cancer immunotherapy of blocking immune checkpoint signaling frequently induces a wide spectrum of irAEs, ranging from skin rash to numerous organ lesions, including life-threatening myocarditis^[24]^. In this context, the toxicity of immune checkpoint inhibitors (ICIs) is mediated through an array of comprehensive mechanisms, including the elevated inflammatory cytokines, up-regulated auto-antibodies, and high activities of T cells against tumor and normal tissue antigens^[25]^. While autoimmunity signs of relevant organ pathology resulting from ICIs need to be mindfully monitored, medical management of these irAEs varies according to individual clinical grades^[24,26]^. Of note, the antibodies against the programmed cell death 1 (PD-1) receptor and its ligand (programmed death ligand 1, PD-L1) cause a lower incidence of any grade irAEs than the antibodies against cytotoxic T-lymphocyte antigen-4 (CTLA-4) do^[9,24]^. Intriguingly, whereas the mechanisms behind various mucocutaneous lesions upon treatment with differential anti-epithelial growth factor receptor (EGFR) are yet to be elucidated, a cellular immunity-based pathogenesis has been proposed^[27]^. As such, to reduce immune network-associated adverse events of the antibody therapy, examining the genetic background of individual patients proves clinically beneficial. For the interest of pharmaceutic research and development to diminish the immunotoxicity, the antibody structure-optimizing strategy comprises full human/humanization and removal of T-cell epitopes through computational prediction and protein engineering^[3,27–28]^.

## Cellular therapy

Over the past two decades, the biomedical landscape has been highlighted by the dramatic emergence of cellular therapy products, such as adaptive immune cells and stem cells, addressing unmet clinical needs of life-threatening illnesses^[4–5]^. In parallel, it is worth noting that the clinical application of these emerging biological products also comes with a high morbidity of irAEs because of bearing numerous cellular antigens and their potentially comprehensive immune mechanisms of action^[5,9]^. For example, chimeric antigen receptor T (CAR-T) cell therapy has achieved curative success in treating certain types of refractory hematological malignancies^[4]^. Unfortunately, the CAR-T cell approach may simultaneously cause a unique profile of severe adverse events upon activating potent immune cells, with cytokine-release syndrome and CAR-T cell-associated encephalopathy, posing major challenges for clinical practice^[5,12,29]^. To deal with these problems, while corticosteroids and the inflammatory cytokine-neutralizing antibodies appear to be helpful for mitigating the toxic effects, optimized CAR-T cells through an improved engineering gene vector of the third generation have been shown to reduce the irAEs without compromising anti-cancer efficacy^[30–31]^. Beyond the hematological indications, the stem cell therapy strategy has been demonstrated to confer clinical benefits for several parenchymal cell damage-caused organ lesions without efficacious treatment, such as myocardial infarction and spinal cord injury^[30]^. Accordingly, to address the immunogenicity issues in those contexts, immune-privileged approaches have been in progress, including mesenchymal stromal cells, autologous induced pluripotent stem cells, and the knockout of major histocompatibility complex (MHC) through gene editing technology^[32–33]^.

## Heparin polysaccharides

Heparin-derived medications have significantly contributed to the management of thrombotic pathology in a wide variety of clinical conditions^[34]^. Meanwhile, concerning the adverse effects of heparin compounds, an immunogenicity-mediated complication termed heparin-induced thrombocytopenia (HIT) should not be ignored^[35]^. Of note, HIT is characterized by distintive features, including platelet factor 4 (PF4) involvement, platelet activation, and elevated antibodies. Accordingly, there is a consensus that the immunogenicity tests for heparin-associated products need to characterize the molecular complex formed by PF4 binding with heparin polysaccharides and others^[7,36]^. In clinical settings, an array of associated risk factors have been observed, including long-term injection of heparin agents, concomitant autoimmune disorders, and surgical inflammation^[37]^. Regarding the source and structure of active pharmaceutical ingredients, bovine heparin appeared to have a higher incidence of HIT than porcine-derived heparin compounds, whereas unfractionated heparin (UFH) was speculated to induce more HIT events than low molecular weight heparin (LMWH) formulations, such as dalteparin and enoxaparin^[36–37]^. A plausible explanation is that LMWH agents have lower molecular weights, compared with UFH, thus being less likely to interact with immune cells and particularly circulating white blood cells^[34,38]^. Moreover, as one of the novel synthetic heparin-like compounds, fondaparinux emerges as a better therapeutic option for indicated patients at HIT risk induced by UFH or LMWH immunogenicity^[39]^.

## Gene medication

In recent years, there has been a remarkable advancement in gene function modulation at the nucleic acid level, which is dramatically translated from basic science into beside in the clinic, such as RNA-based approaches^[40–41]^. Impressively, mRNA vaccines against coronavirus 2019 (COVID-19) have been developed as an outstanding innovative medication to confer a prophylactic efficacy with a therapeutic benefit in alleviating clinical severity of the disease^[41–42]^. Nonetheless, it has also been noted that mRNA vaccines can induce untoward immunogenicity that leads to rare adverse events. In this regard, the emerging myocarditis and immune thrombocytopenia with subcutaneous hemorrhage upon the vaccination may require corticosteroid hormone treatment^[43–44]^. Accordingly, to mitigate the immuno-toxicity of mRNA agents, improvement of the delivering techniques has been proposed, in addition to nucleotide sequence modifications, such as 5′-end capping, and the selected point methylation^[42,45]^.

At the DNA level, the recombinant adeno-associated virus (AAV) vector system has emerged as the most popular platform for delivering gene therapy^[46]^. To date, several AAV-based bio-pharmaceutical products have been approved to enter the medical market to address certain unmet clinical needs, particularly single gene defect-caused diseases, such as hereditary lipoprotein lipase deficiency and spinal muscular atrophy type 1^[46–47]^. Unfortunately, anti-AAV antibodies are identified in the majority of human populations, even prior to the treatment initiation with an increase to higher levels afterward^[4,46]^. On the other hand, it is noted that cellular immunity involving CD4^+^ and CD8^+^ T lymphocytes is activated upon the AAV gene therapy, leading to hepatocyte damage, liver failure, and systemic inflammation in worse scenarios^[47–48]^. Moreover, COVID-19 vaccines based on adenovirus vectors have been associated with a rare adverse event of immune thrombotic thrombocytopenia^[49–50]^. To address these complicated immunogenicity issues, ongoing approaches focus on optimizing the engineering of the capsid variants to evade pre-existing ADA^[45]^, and improving tissue-selective gene delivery to avoid off-target organ involvement^[47,51]^. Of note, there is an escalating interest in applying AAV gene therapy to treat certain genetic disorders of the eyes, because the immune-privileged location and minor dosing of viral vectors needed for the therapeutic purpose therein^[46,52]^.

## Conclusions and perspectives

Addressing untoward immune effects of medications has been a focus for over half a century, and this effort has intensified in recent years with the evolving therapeutic landscape and the emergence of novel biological products (***Table 1***). As extremely rare scenarios, allergic events to small chemical compounds are currently stimulating the development of contemporary targeted pharmaceutical agents^[12]^. It should be noted that *in vitro* synthetic or genetic engineering-expressed peptide/protein products of human sequences have substantially minimized the immunogenicity, compared with those isolated from animal sources. In contrast, checkpoint-inhibiting antibodies are associated with a remarkably higher incidence of immune-mediated adverse reactions^[24,27]^. Whereas certain autogenic cell manipulation-based therapies have achieved unique clinical successes, allogenic cell approaches often encounter challenges with immune rejection^[29,31]^. Interestingly, although the advanced molecular modifications and delivering materials have dramatically diminished the immunotoxicity of gene medications, few emerging biological agent-induced severe adverse events are yet to be deciphered^[43–44]^. Therefore, according to the relevant regulatory guidelines, immunogenicity risks of biotherapeutic products must be assessed throughout their whole life cycles^[3–4,53]^.

**Table 1:** Immune toxicology profile of medication

Categories	Examples	Pathogenesis	Mitigation	References
	Penicillin	Hapten model, ADA/IgE, and histamine release	Structural modification and epimer removing	
Small molecule	Sulfonamide			[1,15–16]
	NSAIDS			
	Insulin	Breaking immune tolerance, and ADA/IgG	Human sequence synthesis/recombination and structural optimization	[18,20–21]
Peptide	Erythropoietin			
	Factor Ⅷ			
	Adalimumab	ADA/IgG, and T-cell activation	Human sequence deimmunization and target differentiation	[3,27–28]
Antibody	Nivolumab			
	Pembrolizumab			
Cellular therapy	CAR-T	T-cell activation Immune rejection	Autogenic source and allogenic MHC deleting	[30–31]
	Stem cell			[32–33]
Heparin	UFH	PF4-heparin complex, and ADA/HIT IgG	Raw material control and processing optimization	[37–39]
	LMWH			
Gene medicine	mRNA agent	ADA/IgG/IgE, T-cell activation, and PF4	Site-specific modification and delivering optimization	[45–46,51]
	Viral formulation			
Abbreviations: NSAIDS, non-steroidal anti-inflammatory drugs; ADA, anti-drug antibody; CAR-T, chimeric antigens receptor-T cell; MHC, major histocompatibility complex; UFH, unfractionated heparin; LMWH, low molecular weight heparin; PF4, platelet factor 4; HIT, heparin-induced thrombocytopenia.				

Looking forward, while ADA, particularly with its neutralizing activity, has been defined as a key parameter to predict potential risks of unwanted immunogenicity, a more comprehensive dissection of the relevant immune modulating network is necessary for the upcoming wave of biological agents^[54]^. In the field of cancer immunotherapy, clinical practice is witnessing an intriguing dynamic, where irAEs may be associated with therapeutic effectiveness^[55]^, which conceivably inspire pharmacovigilance services to more thoughtfully evaluate benefits over risks^[24]^. Moreover, advanced technique platforms are innovatively developed to mitigate the emerging immune-mediated toxicities of new-generation biological medications, such as the MHC gene deletion for universal CAR-T cells^[56]^ and the sequence site-specific modifications for mRNA vaccines^[45]^. Hence, taking advantages of the cutting-edge scientific progress, extraordinary protein/cell/gene-based products with breakthrough efficacy are emerging to address unmet medical needs, and may simultaneously be complicated with novel untoward immune effects; for the latter challenges, relevant mitigating measures are evolving upon insights into advanced pharmaceutic processing arts and human host biology^[3,57]^.
